# Should octogenarians and people aged 90 years and over be treated separately in studies of stroke? An evaluation of national audit data

**DOI:** 10.1093/ageing/afaf164

**Published:** 2025-06-11

**Authors:** Meabh Kelly, Joan McCormack, Olga Brych, Peter J Kelly, Tim Cassidy, Ronan Collins, Joseph A Harbison

**Affiliations:** Department of Medical Gerontology, Trinity College Dublin, Dublin, Ireland; St James’s Hospital Mercer’s Institute for Successful Ageing, Dublin, County Dublin, Ireland; National Office of Clinical Audit, Irish National Audit of Stroke, Dublin, County Dublin, Ireland; National Office of Clinical Audit, Irish National Audit of Stroke, Dublin, County Dublin, Ireland; National Office of Clinical Audit, Irish National Audit of Stroke, Dublin, County Dublin, Ireland; Department of Neurology, University College Dublin, Dublin, Leinster, Ireland; National Office of Clinical Audit, Irish National Audit of Stroke, Dublin, County Dublin, Ireland; Geriatric Medicine, St Vincent’s University Hospital, Dublin, Leinster, Ireland; National Office of Clinical Audit, Irish National Audit of Stroke, Dublin, County Dublin, Ireland; Medical Gerontology, Trinity College Dublin, Dublin, Leinster, Ireland; Health Service Executive, National Clinical Programme for Stroke, Dublin, County Dublin, Ireland; Department of Medical Gerontology, Trinity College Dublin, Dublin, Ireland; National Office of Clinical Audit, Irish National Audit of Stroke, Dublin, County Dublin, Ireland; Stroke Medicine, St James’s Hospital Mercer’s Institute for Successful Ageing, Dublin, County Dublin, Ireland

**Keywords:** ageing, ischaemic stroke, intracerebral haemorrhage, older people

## Abstract

**Introduction:**

Population studies frequently use ≥80 years for defining ‘very old’ but as mean life expectancy frequently exceeds 80 years internationally, this may no longer be appropriate. Those ≥90 years now represent a significant proportion of stroke patients. We examined national data to examine the differences between those 80–89 years and those 90+ years.

Methods Data from the Irish National Audit of Stroke (2017–22 inclusive), including demographic, admission and outcome data, including prestroke and discharge modified Rankin Scores (mRS), were analysed. Proportional data were analysed using Chi-square statistics.

**Results:**

Data on 26 829 individual stroke events were analysed of which 7329 (27.3%) were in people 80–89 years; 52.8% were women. 1708 events occurred in people ≥90 (6.4%); 70.0% were women. 73.7% of those 80–89 years had mRS < 3 prestroke vs 51.3% of those ≥90 (*P* < .001). In hospital mortality for people ≥90 was higher (26.8% vs 17.4% *P* < .001) and they were less likely to have mRS < 3 at discharge (17.0% vs 35.8% *P* < .001). Proportion of haemorrhagic stroke was significantly lower in those ≥90 (15.3% vs 12.9% *P* = .015). Only one haemorrhage was reported amongst 31 people ≥100 years. The proportion of atrial fibrillation (AF) detected following stroke was not significantly different (≥90 years: 33.9%, 80–89 years, 32.4% *P* = .38).

On logistic regression, nonrecovery to independence (mRS >2) in those ≥90 was associated with prestroke mRS, haemorrhagic stroke, AF and being thrombolysed.

**Conclusion:**

There are differences in profiles and outcomes between the groups, and it is now more appropriate to consider them separately.

## Key points

- The population ≥90 years experiencing acute stroke has characteristics that distinguish it from even those in their eighties.
- The rate of intracerebral haemorrhage in patients ≥90 years was lower, partially due to lower anticoagulant use.
- Independent recovery ≥90 years was associated with prior disability, stroke type, and AF but not with age or sex.

## Introduction

The life expectancy of most populations is increasing. Life expectancy at birth in much of Europe and North America exceeds 80 years [1–3]. Demographic changes have resulted in increasing proportions of many countries’ populations being considered ‘very old’. In many prevalence studies of stroke, this distinction is applied to those over 80 or occasionally over 85 years [4–6]. With an increasing population living to and beyond eighty we are seeing a larger number of people ≥90 years who may be better considered of ‘advanced old age’. In the 21st century many of those living into their ninth or 10th decades retain substantial physiological and cognitive reserve. Frailty is a major determinant of recovery from major illness and although methodology for defining frailty varies, a study by Gale and colleagues from the English Longitudinal Study of Ageing [4], showed that the prevalence of frailty in people in their nineties is double that in their eighties. This finding is supported by recently published data from Ireland [5].

Stroke affects all ages but is particularly prevalent in old age [6, 7]. The median age of stroke in the UK and Ireland is about 74 years [8, 9]. Reflecting the fact that studies frequently combine people ≥80 or ≥85 years as the oldest cohort [6, 10, 11], there is little published on differences or inconsistencies in characteristics between people experiencing stroke in their 80s and those ≥90 years. When combining age groups, studies typically consider those <50 years separately as young stroke and also combine those 65–79 years together. This appears done in preference to classification by decade of because of small numbers that may be available in groups at extremes of age.

Given the differences in proportion of frail patients in the population ≥90 years, the increase in prevalence of conditions like Cerebral Amyloid Angiopathy [12] (CAA), variation in the treatment of risk factors, such as the use of anticoagulation [13] and potential difference in administration of interventional therapies, we hypothesised that the characteristics of stroke patients ≥90 years may be substantially different to those 80–89 years and enough to justify their treatment as a separate cohort of patients. Data on stroke have been collected by the Irish National Audit of Stroke (INAS) on patients of all ages for more than a decade and much of the stroke care in Ireland is provided by teams led by stroke physicians trained in Geriatrics and Internal Medicine, a potential advantage when caring for and studying older patients [14].

## Methods

Data on ischaemic stroke (IS) and primary Intracerebral haemorrhage (PICH) from INAS [9, 15] were analysed as part of a cross-sectional, cohort study. Data on sub-arachnoid haemorrhage are not systematically collected. INAS is a dataset routinely collected by clinical stroke teams in all hospitals admitting at least 25 stroke patients per annum [15]. The data are collected into a national online database where it is merged with routinely collected demographic, process and outcome data from the Hospital In-Patient Enquiry. All hospitals involved in the study have geriatricians involved in stroke care [14] and the practise of Comprehensive Geriatric Assessment is widely implemented [16]. As the older cohort of stroke patients is heavily weighted towards women for reasons of increased survival and later stroke onset, data for the entire population were also analysed to look for evidence of discrepancies in care related to sex. Data for the population 80–89 years were then compared with that of people ≥90 years.

Data for age, sex, length of stay and mortality and data for disability, as measured by modified Rankin score (mRS) were collected for prestroke and discharge. Data for indices of care, including prestroke detection of atrial fibrillation (AF) and utilisation of anticoagulation for secondary prevention of cardio-embolism, were collected in addition to data on Stroke Unit Admission and Thrombolysis rate.

Data were analysed using statistical software (SPSS v29) and Microsoft Excel. Comparisons for continuous and quantitative data were made using Student’s t tests and for proportional data using Pearson’s Chi-square statistics and regression analyses were performed. As the dataset is fundamentally designed for the purposes of quality improvement, a number of covariates that may be available in an observational study are not collected, e.g. comorbidities, body weight or NIH Stroke Scale (NIHSS) impairment score. This absence potentially affects regression analyses. Some data points, notably prestroke and discharge mRS were not collected on a minority of patients, this data are presented in the results tables. Although all data were anonymised prior to analysis, ethical approval was obtained for the study from the joint University Hospital institutional ethics committee (ref 3989) to comply with institutional practise for INAS for data use outside standard outcome reporting. INAS is a statutorily funded organisation and no external sources of funding were used for this study.

## Results

Data on 26 829 individual stroke incidents were collected in the 6 years 2017–22 inclusive. Of these, 23 033 (85.9%) were IS and 3796 were PICH. Eleven thousand five hundred seven (42.9%) were women. Median age of stroke onset was 72 in men (IQR 62-80) and 78 in women (IQR 6-85) (*P* < .0001). The proportion of PICH was higher in women (15.6% vs 13.1%. *P* < .001, Chi-square). There were no significant differences in management identified between sexes other than a significant difference in the proportion of people who had their AF identified and anticoagulated prestroke (Table 1).

**Table 1:** Demographic details and sex differences identified in the INAS dataset 2017–22.

	Total		Male		Female		
	*n* (% of total)	Median	*n* (%)	Median	*n* (%)		
Length of stay (median days)	26 829	9	15 322 (57.1)	8	11 507 (42.9)	9	
Age (median years)	26 829	74	15 322 (57.1)	72	11 507 (42.9)	78	
<60 years (% of total population)	4688 (17.5)		3176 (20.7)		1512 (13.1)		<0.0001
≥80 years (% of total population)	9038 (33.7)		3971 (25.9)		5067 (44.0)		<0.0001
Prestroke mRS (% population)	24 260 (90.4)	0	13 866 (90.4)	0	10 394 (90.3)	0	
Prestroke mRS <3 (%)	20 499 (84.5)		12 332 (88.9)		8167 (78.6)		<0.0001
Discharge mRS available (% population)	24 067 (89.7)		13 725 (89.6)		10,342 (89.8)		
Discharge mRS <3 (%)	12 804 (53.2)		8064 (58.8)		4740 (45.8)		<0.0001
Discharge mRS 6: mortality rate. (%)	2817 (11.7)		1374 (10.0)		1443 (14.0)		<0.0001
Ischaemic stroke (% population)	23 034 (85.9)		13 318 (86.9)		9716 (84.4)		<0.0001
AF known pre stroke (% ischaemic stroke)	3134 (13.6)		1936 (14.5)		1198 (12.3)		<0.0001
AF known prestroke (% haemorrhagic stroke)	507 (13.3)		299 (14.9)		208 (11.6)		0.007
AF known and anticoagulated prestroke (% known AF pre-ischaemic stroke)	2552 (81.4)		1602 (82.7)		950 (79.3)		0.015
AF known and anticoagulated prestroke (% known AF pre-haemorrhagic stroke)	461 (90.9)		271 (90.6)		190 (91.3)		0.78
AF detected as inpatient, known and new. (% of total)	5920 (22.1)		3334 (21.7)		2586 (22.5)		0.16
Thrombolysis rate (% Ischaemic stroke)	2407 (10.4)		1366 (10.3)		1041 (10.7)		0.26
Admitted to stroke unit (%)	18 990 (70.8)		10 818 (70.6)		8172 (71.0)		0.46

There were 7329 events in people aged from 80–89, representing 27.3% of the total population of which 3871 (52.8%) were in women. There were 1708 events in people aged ≥90 years, representing 6.4% of the population and 1196 of these were in women (70.0%) (Table 2). There were 1963 strokes in people <50 years, 7.3% of the total, a group typically considered distinct in published studies. The population ≥90 years were substantially more disabled at baseline. Only 51.3% were categorised as independent (mRS <3) prestroke compared with 73.7% of those 80–89 years (*P* < .001). In-hospital mortality for the older age group was significantly higher (26.8% vs 17.4% *P* < .001) and the ≥90 population were less likely to be independent at discharge (17.0% vs 35.8% *P* < .001), although 33.1% of those over 90 admitted with an mRS <3 were discharged with an mRS <3, suggesting an ability to ambulate and complete activities of daily living independently.

**Table 2:** Differences in the INAS dataset between population aged 80–89 years and those age 90 years and older.

	Total population		80–89 years		≥90 years		Difference between 80–89 years and ≥90 years
	*n* (% of total)	Median	*n* (%)	Median	*n* (%)		
Length of stay (median days)	26 829	9	7329 (27.3)	11	1708 (6.4)	11	
Age (median Years)	26 829	74	7329 (27.3)	84	1708 (6.4)	92	
Prestroke mRS (% population)	24 260 (90.4)	0	6496 (88.6)	1	1512 (88.5)	1	0.89
Prestroke mRS <3 (%)	20 499 (84.5)		4788 (73.7)		776 (51.3)		<0.0001
Discharge mRS available (% population)	24 067 (89.7)		6475 (88.3)		1538 (90.0)		.046
Discharge mRS <3 (%)	12 804 (53.2)		2316 (35.8)		412 (17.0)		<0.0001
Discharge mRS 6 (%)	2817 (11.7)		1130 (17.4)		1542 (26.8)		<0.0001
Ischaemic stroke (% population)	23 034 (85.9)		6210 (84.7)		1487 (87.1)		.015
Haemorrhagic stroke	3795		1119 (15.3)		221 (12.9)		
AF known pre stroke (% ischaemic stroke)	3134 (13.6)		1233 (19.8)		278 (18.7)		0.3
AF known pre stroke (% haemorrhagic stroke)	507 (13.3)		223 (19.9)		45 (20.3)		0.9
AF known and anticoagulated prestroke (% known AF pre-ischaemic stroke)	2552 (81.4)		1029 (83.5)		197 (72.9)		<0.0001
AF known and anticoagulated prestroke (% known AF pre-haemorrhagic stroke)	461 (90.9)		205 (91.9)		40 (88.9)		0.56
AF detected as inpatient, known and new. (% of total)	5920 (22.1)		2374 (32.4)		579 (33.9)		0.23
Thrombolysis rate (% ischaemic stroke)	2407 (10.4)		608 (9.8)		139 (9.3)		0.60
Admitted to stroke unit (%)	18 990 (70.8)		5216 (71.2)		1179 (69.0)		0.08

The proportion of PICH was lower in the ≥90 years group than in the 80–89 group 12.9% vs 15.3% (*P* = .015 Chi-square). This appeared to be due in part to a lower level of anticoagulation in those ≥90 (≥90 years: 13.6%, 80–89 years, 16.4% *P* = .003). This difference was mainly due to a significant drop in the proportion of women ≥90 receiving anticoagulation (Men ≥90 years: 15.2%, 80–89 years: 14.2% *P* = .57. Women ≥90 years 12.0%, 80–89 years 16.2% *P* = .002), women being the majority of the older population. This was not associated with a significant difference in the proportion of women with known AF at stroke onset (Men ≥90 years with known AF: 23.4%, 80–89 years: 21.9% *P* = .44. Women ≥90 years with known AF: 17.0%, 80–89 years: 18.1% *P* = .38) and the proportion of men and women aged 90+ who were not anticoagulated for known AF was similar (Men 25.8%, Women 27.1% *P* = 0.08). Only one stroke in those ≥100 years (*n* = 31) was haemorrhagic.

The proportion of both groups who had AF detected following stroke was not significantly different (≥90 years: 33.9%, 80–89 years, 32.4% *P* = .38) (Table 2). Those with IS with known AF were significantly less likely to have been anticoagulated prestroke in the older group (83.5% vs 72.9% *P* < .001). There were no significant differences seen in the rate of admission to stroke unit (71.2% vs 69.0%, *P* = .08) or in thrombolysis rate (9.8 vs 9.3%, *P* = .6) between younger and older groups.

On logistic regression, nonrecovery to independence (mRS >2) in the over 90s was associated with increasing prestroke mRS, PICH, presence of AF and being thrombolysed (Table 3). Being admitted to a stroke unit was also associated with a worse functional outcome. No significant association was found for either increasing age within the group or with sex.

**Table 3:** Logistic regression: predictors of nonrecovery to independence (mRS >2) in those ≥90 years.

	β coefficient	SE	OR	CI	*P*
Age	0.043	0.024	1.044	0.996–1.044	.08
Sex	−0.036	0.140	0.964	0.733–1.260	.79
Prestroke mRS	0.115	0.043	1.112	1.031–1.221	.007
Haemorrhagic stroke	1.124	0.166	3.078	2.223–4.262	<.001
Thrombolysed	−0.853	0.206	0.426	0.285–0.638	<.001
Atrial fibrillation present	0.101	0.037	1.106	1.029–1.189	.007
Admitted to stroke unit	0.450	0.136	1.568	1.201–2.047	<.001

Linear regression was performed using mRS on discharge as a continuous variable (Table 4). We again found it significantly associated with increasing prestroke mRS, haemorrhagic stroke, length of stay and being thrombolysed. No significant association was again found with age or sex. When the regression was performed again (Table 5) following the removal of prestroke mRS from the analysis, both increasing age and female sex were found associated with increasing discharge mRS scores, suggesting that two factors are substantially confounded by level of prestroke disability.

**Table 4:** Linear regression analysis was conducted to determine associations of level of disability on discharge indicated by the mRS (0–7) in those ≥90 years.

	β coefficient	SE	OR	CI	*P*
Age	.025	.015	1.284	1.248–1.321	.103
Sex	−.008	.084	0.992	0.841–1.171	.924
Prestroke mRS	.429	.026	1.536	1.459–1.617	<.001
Stroke type	.825	.113	2.282	1.828–2.849	<.001
LOS	.007	.002	1.007	1.003–1.011	<.001
Thrombolysed	−.487	.138	0.614	0.469–0.806	<.001
Atrial fibrillation present	−.125	.077	0.882	0.759–1.026	.104
Admitted to stroke unit	.035	.087	1.036	0.873–1.229	.684

The model explained 21% of the variance (R^2^ = 0.21, SE 1.44).

**Table 5:** When prestroke Rankin score is removed from the model the R2 increased to 0.24 (SE 1.577) and age and sex become significant associations.

	β coefficient	SE	OR	CI	*P*
Age	.055	.016	1.056	1.024–1.090	<.001
Sex	.203	.091	1.225	1.024–1.465	.026
Prestroke mRS	.886	.124	2.426	1.902–3.093	<.001
Haemorrhagic stroke	.007	.002	1.007	1.003–1.011	<.001
Thrombolysed	−.475	.150	0.622	0.463–0.835	.002
Atrial fibrillation present	−.192	.084	0.825	0.700–.973	.022
Admitted to stroke unit	.182	.094	1.200	.100–1.439	.054

## Discussion

The population experiencing stroke aged ≥90 years has characteristics that distinguish it from those 10 years younger. The rate of prestroke disability is substantially higher and outcomes are substantially worse despite similar access to organised care and intervention. Almost one-third of those people aged 90 and over who were independent prestroke, remained independent at discharge from hospital. Over the age of 90, the rate of AF-associated stroke was not higher than the population in their eighties and the rate of PICH appeared to be lower, in part, to less use of oral anti-coagulant therapy. People in their eighties represented more than one quarter of all strokes in the database and people ≥90 represented more than 5%, similar in proportion to those<50 years.

There are limitations to this form of observational study. Data are collected as a routine by clinicians in hospital-based stroke teams and there is incomplete data. The proportion of missing modified Rankin data was very similar in all age groups and the characteristics of the population with missing data was similar. Another limitation is that INAS is hospital based and only collects data from acute general hospitals with stroke services. It is likely that older patients experiencing massive stroke in long-term nursing facilities, or even at home, or those who have significant other comorbidities may not be referred into hospital as a decision by their families or family doctor. Similarly, people may choose not to refer in people with only minor impairments if they occur in the context of other comorbidity like dementia or frailty. A previous study in North Dublin in Ireland found that 9.5% of people with acute stroke did not present to hospital [17] but that proportion is likely to be higher both in the age group under evaluation and in non-urban areas with less convenient access to hospitals.

INAS, in common with most audits, does not collect data on stroke subtype or aetiology beyond whether a stroke is ischaemic or haemorrhagic. Our finding that the population ≥ 90 years were less likely to be anticoagulated for known AF prior to admission may have contributed in part to their significantly lower rate of intracerebral haemorrhage. A number of previous studies have shown an attenuated increase or even a decrease in intracerebral haemorrhage in a combined cohort ≥85 years [7]. This may seem unexpected however the most important risk factor in haemorrhagic stroke is hypertension and studies have failed to consistently show substantial survival advantage to treating hypertension in people ≥90 years [18]. It may be that this survivor group effect and the lower rate of anticoagulation contributed to the lower incidence of haemorrhagic stroke in the population. In contrast the prevalence of CAA, the commonest cause of haemorrhagic stroke in nonhypertensives, has been reported as high as 43%–58% in those ≥90 years [7]. However, people with CAA remain more likely to experience ischaemic than haemorrhagic stroke. What is impossible to determine from this data is if the lower use of anticoagulation in these subjects was warranted or was associated with a higher rate of cardioembolic stroke. A specific, prospective study considering etiological subtype and impairment scores in this age group looking at risks including sex would be necessary for confirmation. Our finding of no significant difference in rate of AF between those in their 80s and ≥90 may imply a similar incidence of cardioembolic stroke. Little comment can be made in respect of stroke location an incidence of lacunar stroke with advancing age above 80 years because studies have typically cohorted these individuals together even in large studies [19, 20].

At the time of this study INAS used prestroke and discharge mRS to measure change in function as this is the primary outcome measure in most stroke intervention studies. We did not record patients’ admission NIHSS. The NIHSS is a measure of impairment rather than disability and prestroke mRS is more frequently used to determine whether patients are fit for interventions such as thrombolysis [21] or thrombectomy [22]. Existing comorbidity can also substantially influence the poststroke NIHSS, which records all neurological deficits, not just those associated with the acute event, and prestroke NIHSS cannot be accurately estimated to identify this. There is also a more complex training process involved in utilising the NIHSS that could not, until recently, be delivered to all staff admitting stroke in Ireland. Prestroke and discharge mRS can be applied by trained stroke service staff who ultimately care for the patients.

As part of our analysis, we found the perhaps surprising result that patients admitted to stroke units had worse outcomes. This has been noted before [13] and is inconsistent with findings of a beneficial effect of organised stroke care in units [23]. We think this is likely to be due to the lack of availability of sufficient stroke unit beds in the system. In the absence of sufficient beds. People with more severe strokes may be prioritised to specialist beds and people with more minor events may be investigated and discharged to outpatient follow-up or rehabilitation without ever having entered a stroke unit. An ongoing recommendation of INAS has been the provision of more stroke unit capacity in the country both through provision of additional beds, including in single rooms and additional capacity in supported discharge teams [9]. There was no evidence that older people experienced a worse outcome in units and our findings support the findings of the Cochrane Collaboration that stroke unit care benefits all subgroups admitted [23].

Published cross sectional and population research has varied in what they consider to be very or extremely old [10]. Most often they have selected 80 years [10, 24] as the determinant though some have used 85 [6]. With the mean life expectancy of many countries now surpassing 80 years, and more than one-quarter of patients falling in the age group 80–90 it is perhaps time to consider people older than this in their own respect as they have substantially different characteristics and may have additional needs in terms of time to recovery, rehabilitation requirements and prevalence of comorbidity and frailty, which will impair recovery [4, 25, 26]. In studies of frailty, incidence and prevalence of increase particularly rapidly in peoples’ eighties [27, 28]. However physical functioning has been found in England and China to be improving in ‘younger’ old age in recent decades [29] and prevalence of frailty in older age has been found to be declining in some cohorts [30]. In terms of intervention, advanced age is no longer itself a contraindication to thrombolysis or thrombectomy [31–33] whereas premorbid disability, as measured by the mRS, often can be. In an operative intervention, vascular surgeons are now far more open to perform carotid endarterectomy in people in their 80s [34, 35] but the number of procedures performed in the population ≥90 years is small both due to perceived operative risk and the limited potential for long-term benefit [36]. Clinical trials in stroke have largely stopped applying upper age limits to inclusion criteria. These limits previously impeded evaluation of and access to effective interventions for older people. For instance, some initial thrombolysis studies [37, 38] excluded people over 80 whereas more recent thrombectomy studies included them and found they had a greater differential benefit to the intervention [39]. The challenge remains that trials typically want to recruit patients with few comorbidities. This eliminates most people in their nineties and many in their eighties. Thus, interventions in very old patients can often be implemented on the basis of sparse-specific evidence and only following post-hoc studies can we get some evidence of their effects in this age group. In determining the specific risks and benefits of interventions in this population we need a combination of more pragmatic trails and a process post implementation evaluation of older populations to confirm their benefit or scale of any additional hazard.

The distinction between 89 and 90 years may be arbitrary but it is a more rational and evidence-based point of delineation to define both very advanced age and likelihood of comorbidity and frailty and with more validity than the transition between 79 and 80 years or 80 and 81 years. With changing demographics, improved physical functioning and the increased use of intervention in people in their eighties it is appropriate to consider this older and enlarging group of those 90 and above separately in analyses of population data and presentation of results of stroke studies. Despite the high level of both prestroke and discharge disability in those ≥90 years, about one-third found independent at admission were still independent on discharge. These may represent a biologically more robust group and they may have proportionally less severe strokes but it indicates that within this population there is little reason to conclude that this group and others of advanced age do not benefit from interventions from thrombectomy to rehabilitation therapies. There may also have been some benefit to these patients in being admitted to services predominantly delivered with the involvement of Geriatricians and with a Comprehensive Geriatric assessment model. As our multivariate analyses have demonstrated, even in people over 90 years, age has a weaker association with outcome than underlying premorbid level of disability.

In conclusion, there appear to be significant differences between stroke characteristics and outcomes in people in their eighties compared with those ≥90 years who represent <20% of the combined group but differ substantially. This acknowledged that there is potential for the oldest, old to still benefit from organised stroke care and to receive appropriate intervention and there are grounds to advocate for more detailed and robust analyses of stroke characteristics, interventions and outcome in older people.

## Data Availability

The data are derived from publicly available INAS data. Data are accessible on application from the National Office of Clinical Audit, Dublin through their website.
